# Extraperitoneally Ruptured, Everted, and Prolapsed Bladder: A Very Rare Complication of Pelvic Injury

**DOI:** 10.1155/2015/476043

**Published:** 2015-08-31

**Authors:** Rufus Wale Ojewola, Kehinde Habeeb Tijani, Olakunle Olaleke Badmus, Abisola Ekundayo Oliyide, Chukwudi Emmanuel Osegbe

**Affiliations:** ^1^Urology Unit, Department of Surgery, College of Medicine, University of Lagos and Lagos University Teaching Hospital, PMB 12003, Idi Araba, Lagos, Nigeria; ^2^Department of Orthopaedics, Lagos University Teaching Hospital, PMB 12003, Idi Araba, Lagos, Nigeria; ^3^Urology Unit, Department of Surgery, Lagos University Teaching Hospital, PMB 12003, Idi Araba, Lagos, Nigeria

## Abstract

Traumatic rupture of the bladder with eversion and protrusion via the perineum is a rare complication of pelvic injury. We present a 36-year-old lady who sustained severe pelvic injury with a bleeding right-sided deep perineal laceration. She had closed reduction of pelvic fracture with pelvic banding and primary closure of perineal laceration at a private hospital. She subsequently had dehiscence of repaired perineal laceration with protrusion of fleshy mass from vulva and leakage of urine per perineum five weeks later. Examination revealed a fleshy mucosa-like mass protruding anteriorly with a bridge of tissue between it and right anterolateral vaginal wall. Upward pressure on this mass revealed the bladder neck and ureteric orifices. She had perineal and pelvic exploration with findings of prolapsed, completely everted bladder wall through a transverse anterior bladder wall rent via the perineum, and an unstable B1 pelvic disruption. She had repair of the ruptured, everted, and prolapsed bladder, double-plate and screw fixation of disrupted pelvis and repair of the pelvic/perineal defect. She commenced physiotherapy and ambulation a week after surgery. Patient now walks normally and is continent of urine. We conclude that the intrinsic urethral continent mechanism plays a significant role in maintaining continence in females.

## 1. Introduction

The overall incidence of urethral injury following pelvic fracture is said to be 5–10% and only one-third of these will have associated bladder injury [1]. Most bladder ruptures are of the extraperitoneal type and are usually confined to the pelvis with or without perineal urinary extravasation [2]. However, traumatic rupture of the bladder as well as complete bladder eversion with protrusion through the perineum is a very rare complication of pelvic injury. We present a case of delayed presentation of traumatic perineal prolapse of completely everted extraperitoneally ruptured bladder following severe pelvic injury.

## 2. Case Presentation

Miss R. B. is a 36-year-old female clerk admitted via the accident and emergency on account of inability to bear weight of five-week duration and continuous leakage of urine five days prior to presentation. Patient was involved in a pedestrian motorcycle accident in which she was hit by a power bike on a trunk C road. There was immediate loss of consciousness lasting about thirty minutes but no craniofacial bleed, vomiting, or seizures. There was bleeding from associated perineal laceration on the right side of the perineum. She was initially taken to a private hospital where she was noticed to have a perineal avulsion exposing the pelvic bone. She had closed reduction of pelvic fracture with pelvic banding and primary repair of perineal laceration in addition to initial fluid, analgesic, and antibiotic therapies. She was also catheterized with catheter draining clear urine following an initial period of total haematuria for about three days. However, she was referred on account of dehiscence of repaired perineal laceration with protrusion of fleshy mass from vulva with associated continuous leakage of urine per perineum. Following the injury, patient lost the sensation or feeling of bladder fullness. There was no history of loin pain, fever, chills, anorexia, nausea or vomiting.

**Figure 1:**
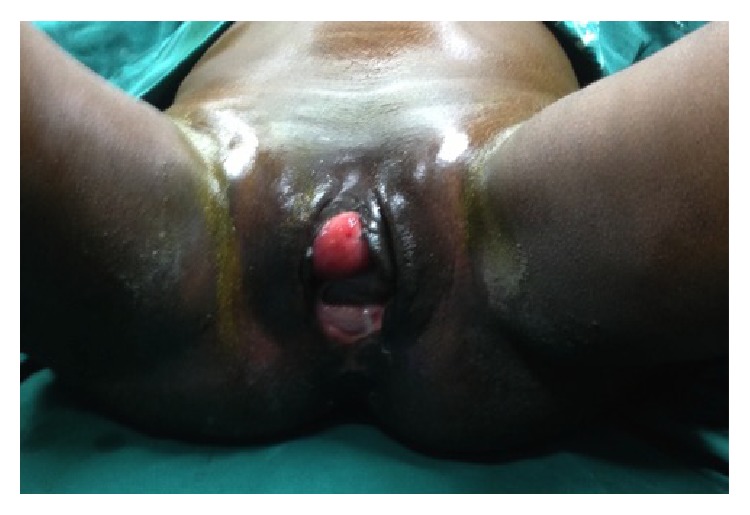
Image showing everted and prolapsed bladder with a bridge of tissue separating it from the vagina at presentation.

**Figure 2:**
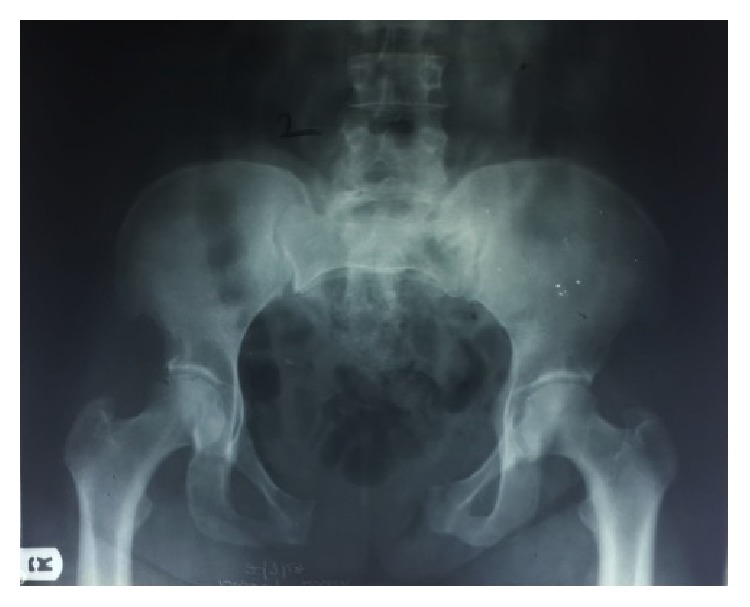
Pelvic X-Ray showing wide pubic diastasis, fractured left upper ischial ramus, and widened sacroiliac joints.

Examination revealed a young lady, conscious and alert, pale, afebrile, not dehydrated, and nil pedal oedema. Abdominal examination was essentially normal. External genitalia examination revealed a fleshy mucosa-like mass protruding per perineum anteriorly measuring about 6 cm × 6 cm with a bridge of tissue containing the labia majora between it and right anterolateral vaginal wall (Figure 1). Upward pressure on this mass reveals the bladder neck and ureteric orifices. Urethra was located in bridge of tissue but deviated laterally to the left. There was a palpable wide pubic diastasis. Posterior vaginal wall was intact and cervix was situated in normal position. Digital rectal examination was essentially normal with no communication between the rectum and posterior vaginal wall. Straight leg raising test was zero degree on the right and 30° on the left. Pelvic X-ray revealed unstable B1 fracture with wide pubic diastasis, fractured left upper ischial ramus, and widening of both sacroiliac joints (Figure 2).

Patient was optimized for surgery and had perineal and pelvic exploration under G.A. Findings at surgery were prolapsed completely everted dome of the bladder wall through a transverse rent in the anterior bladder wall about 1.5 cm above the bladder neck, laterally deviated urethra to the left, pelvic disruption with approximately 10 cm symphyseal separation, shear injury to the body of the right pubis, and a big defect between the right anterolateral vaginal wall and disrupted pubis symphysis.

**Figure 3:**
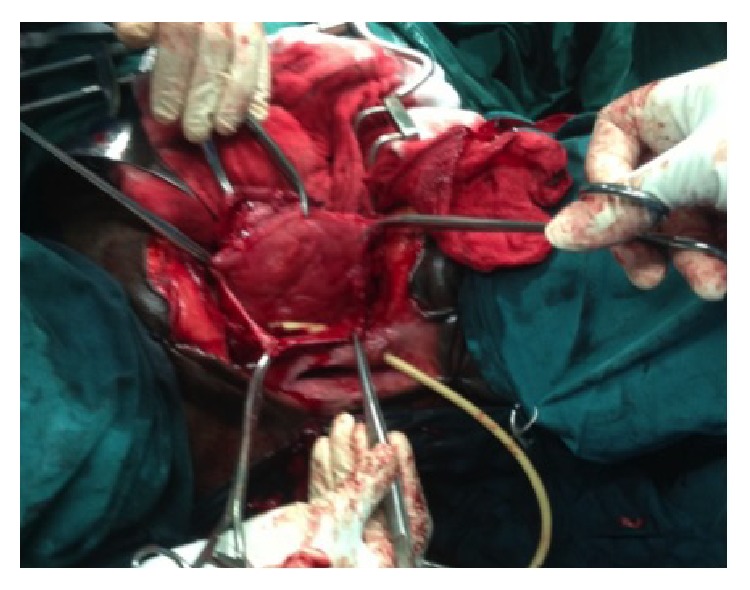
Image showing lacerated bladder edges after reduction of the everted and prolapsed bladder mucosa.

**Figure 4:**
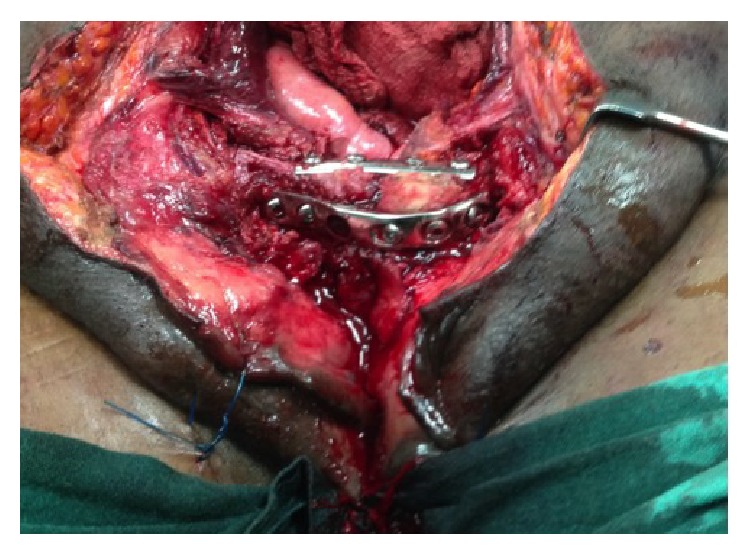
Image showing plated pelvic fracture after reduction and repair of bladder and pelvic floor.

She had repair of the ruptured everted and prolapsed bladder, double-plate and screw fixation of the disrupted pelvic disruption, and repair of the perineal defect in layers (Figures 3 and 4). Urethral catheter was discontinued about three weeks after surgery. Patient had irritative symptoms initially which subsided with the use of Tolterodine and was fully continent of urine after the surgery. She commenced physiotherapy and ambulation with walking frame and now walks normally.

## 3. Discussion

Pelvic fractures are a marker of severe posttraumatic injury and are associated with intra-abdominal and urogenital injuries in 15%–20% of patients [3]. The most commonly injured organ in pelvic fractures is the posterior urethra (5.8%–14.6%), followed closely by the liver (6.1%–10.2%) and the spleen (5.2%–5.8%). The bladder and bladder neck are frequently involved, and injury to these structures needs to be identified and included in the equation of the surgical strategy. Pelvic fracture injury with a bladder rupture is an excellent marker for severe trauma and approximately 90% of patients with a traumatic bladder rupture have an associated pelvic fracture [4].

The commonest injury in females with pelvic fracture injury is complete urethral separation at all levels but most often noted at the proximal bulbar and bladder neck in about 84% of cases [5]. Isolated bladder injury with an intact urethra is a rare occurrence. Partial or complete eversion of the bladder is very rare and is considered a rarer condition in humans compared with animals occurring more commonly in mares [6] and cattle [7]. Only three cases of complete bladder eversion were reported before 1983 when Underhill and Altaffer [8] reported their own case. The main reasons for bladder eversion in these animals have been reported as severe straining/difficulty in late pregnancy or during the early postpartum period, an increase in intra-abdominal pressure, a short and wide urethra in mares, and predisposition to eversion due to hypocalcaemia [6, 7]. In humans, the mechanism is poorly understood but two mechanisms have been proposed. The first is widening of the urogenital hiatus, which can result in pulling of the bladder base and posterior urethra away from the pubic bone. The second is that vaginal prolapse can cause obstructed voiding with straining which starts the process of inversion of the bladder through the urethra [9]. Some earlier reports mentioned complete transurethral inversion of bladder occurring during labour [10] and few reported as complication of vesicovaginal fistula [11]. Senility, obesity, and multiple labours could be possible predisposing factors to eversion and prolapse of bladder. Other reported causes of bladder eversion and prolapse in human were pulling out of Foley's catheter [12] and bladder eversion concurrent with uterine prolapse [13] and primary adenocarcinoma [14]. Trauma as a cause of bladder eversion and prolapse as seen in this case is not widely reported. We could not find in the literature search any of such occurrences secondary to severe pelvic injury. However, isolated bladder rupture or tear without prolapse is a common complication of high-energy events [15].

The diagnosis of bladder prolapse with or without eversion is clinical. It occurs exclusively in females and usually manifests with protrusion of the fleshy mass through the vagina or vulva with continuous leakage of urine. The diagnosis is also straightforward and almost immediate in cases secondary to common causes of this condition. Diagnosis was however delayed for about five weeks in our patient due to unusual aetiology. The diagnosis could have been missed initially by the attending physician who probably repaired the perineal laceration over the prolapsed organ with the pathology becoming evident after dehiscence of the repaired perineal laceration. In addition, associated laceration as we saw in this case is not usually associated with other causes of this condition other than trauma. Furthermore, the protrusion of bladder in typical bladder eversion is usually through the urethra and vagina. In our patient, there was a bridge of tissue between the prolapsed organ and the right anterolateral wall of the vagina (Figure 1). The everted bladder with the folding of the fundus over the trigone and prolapse through the lacerated edge allowed a small space for urine to collect from the two ureteric orifices and eventual drainage through the inserted urethral catheter. This space, which was located in the most dependent part of the bladder, facilitated effective bladder drainage via the inserted urethral catheter. This probably prevented the urinary leakage from the everted edge as patient maintained a supine position throughout her stay on admission from the referral hospital. The everted and prolapsed bladder mucosa also makes the catheter invisible until a thorough examination was carried out at presentation in our facility. The continuous leakage of urine noted weeks later might be due to blockage of the catheter and eventual urine leak from dehisced wound.

Even though the diagnosis of bladder prolapse and eversion is straightforward, this condition must be differentiated from urethral prolapse, prolapse of a ureterocoele, prolapse of redundant bladder mucosa, and a polypoid tumor of the urethra. Intravenous urography and cystoscopy may be very useful in differentiating this condition from the differential diagnosis. However, they are not routinely recommended especially when it occurred following trauma.

The treatment of bladder eversion and prolapse can be conservative or surgical. Manual reduction under general anaesthesia is possible and successful in a number of cases. However, the treatment of the underlying predisposing factor is an important aspect of management. Successful treatment of cases secondary to severe pelvic trauma with disruption of integrity of the pelvic structures requires operative treatment involving combined team of urologists and orthopaedic surgeons as demonstrated in this case. Repair or closure of lacerated anterior wall was necessary after reduction of the eversion followed by reduction and reconstruction of the muscular and bony component of the pelvis in this case. There may be need for surgery for incontinence later for patient with urinary incontinence after the initial surgical reduction [16].

One of the reported complications of this condition is bilateral hydronephrosis and acute renal failure. These usually result from ureteral kinking in complete eversion. A preliminary diversion by nephrostomy or ureteral stenting may be required in these cases to correct azotaemia prior to definitive procedure. This has been reported from complete eversion through a VVF [11]. A case of concurrent adenocarcinoma in the thickened wall of the everted urinary bladder has also been previously documented in the everted bladder [14].

Urinary incontinence following the repair of posttraumatic prolapsed bladder is a likely complication because of disruption of pelvic supportive structures, which plays significant role in maintaining continence. The arcus tendinous fascia pelvis is a condensation of connective tissue, which extends bilaterally from the inferior part of the pubic bone along the junction of the fascia of the obturator internus and levator ani muscle group to near the ischial spine. This tissue provides secondary support to the urethra, bladder neck, and bladder base. Defects in this tissue are believed to result in cystocele development and urethral hypermobility with urinary incontinence. The primary support to this area and the entire pelvic floor is believed to be the levator ani muscle complex. At rest, the constant tone mediated by slow-twitch muscle fibers is thought to constitute the major supportive mechanism. Even though this muscular support was destroyed and lost in this patient, urinary continence was maintained after the repair. This points to the complex nature of urinary continence and the significant role an intact urethral striated muscle component plays in female continent mechanism.

## 4. Conclusions

The bladder and bladder neck are frequently involved in severe pelvic injury. Injuries to these structures need to be identified and included in the equation of the surgical strategy. Although extraperitoneal bladder rupture is usually treated conservatively, concomitant bony injury, eversion, and prolapse of the pelvic viscera require surgical repair. A combined team of urologists and orthopaedic surgeons best accomplished this repair.
